# Quantitative breast density analysis to predict interval and node-positive cancers in pursuit of improved screening protocols: a case–control study

**DOI:** 10.1038/s41416-021-01466-y

**Published:** 2021-06-24

**Authors:** Elizabeth S. Burnside, Lucy M. Warren, Jonathan Myles, Louise S. Wilkinson, Matthew G. Wallis, Mishal Patel, Robert A. Smith, Kenneth C. Young, Nathalie J. Massat, Stephen W. Duffy

**Affiliations:** 1 Department of Radiology, University of Wisconsin-Madison School of Medicine and Public Health, E3/311 Clinical Science Center, Madison, WI USA; 2 National Co-ordinating Centre for the Physics of Mammography (NCCPM), Medical Physics Department, Royal Surrey County Hospital, Guildford, UK; 3 Centre for Cancer Prevention, Queen Mary University of London, Wolfson Institute of Preventive Medicine, London, UK; 4 Oxford Breast Imaging Centre, Churchill Hospital, Oxford, UK; 5 Cambridge Breast Unit and NIHR Cambridge Biomedical Research Centre, Cambridge University Hospitals NHS Trust, Cambridge, UK; 6 Scientific Computing, Medical Physics Department, Royal Surrey County Hospital, Guildford, UK; 7 American Cancer Society, Atlanta, GA USA

**Keywords:** Breast cancer, Risk factors

## Abstract

**Background:**

This study investigates whether quantitative breast density (BD) serves as an imaging biomarker for more intensive breast cancer screening by predicting interval, and node-positive cancers.

**Methods:**

This case–control study of 1204 women aged 47–73 includes 599 cancer cases (302 screen-detected, 297 interval; 239 node-positive, 360 node-negative) and 605 controls. Automated BD software calculated fibroglandular volume (FGV), volumetric breast density (VBD) and density grade (DG). A radiologist assessed BD using a visual analogue scale (VAS) from 0 to 100. Logistic regression and area under the receiver operating characteristic curves (AUC) determined whether BD could predict mode of detection (screen-detected or interval); node-negative cancers; node-positive cancers, and all cancers vs. controls.

**Results:**

FGV, VBD, VAS, and DG all discriminated interval cancers (all *p* < 0.01) from controls. Only FGV-quartile discriminated screen-detected cancers (*p* < 0.01). Based on AUC, FGV discriminated all cancer types better than VBD or VAS. FGV showed a significantly greater discrimination of interval cancers, AUC = 0.65, than of screen-detected cancers, AUC = 0.61 (*p* < 0.01) as did VBD (0.63 and 0.53, respectively, *p* < 0.001).

**Conclusion:**

FGV, VBD, VAS and DG discriminate interval cancers from controls, reflecting some masking risk. Only FGV discriminates screen-detected cancers perhaps adding a unique component of breast cancer risk.

## Background

The aim of stratified, or risk-based, breast cancer screening [1–5] is to optimise the balance of benefits of early cancer detection and mortality reduction with the harms of false-positive mammograms, benign biopsies, and overdiagnosis [4]. However, risk-based protocols may eliminate screening benefits for some women [6, 7] and increase complexity, with questionnaires, blood draws, and counselling, thereby potentially detracting from the performance of an age-based screening programme. To preserve or improve effectiveness, a stratified screening programme needs to maintain or decrease the incidence of advanced (i.e. node-positive) and interval cancers, those tumours most likely to be clinically significant. Ideally, stratified protocols would personalise mammography initiation, screening interval, and supplemental screening with other modalities in order to decrease advanced and interval cancers, while maintaining low rates of false positives. This programme would ideally decrease mortality from breast cancer in all women regardless of risk.

Breast density (BD) reflects the amount of glandular and fibrous connective tissue compared with the amount of fatty tissue in the breasts, as seen on a mammogram. BD has three attributes that support use in stratification of population screening. First, increased BD, conditional on age and body mass index (BMI), is a strong risk factor for breast cancer [8]; second, high levels of BD are associated with lower sensitivity of mammography due to masking, i.e., when dense breast parenchyma obscures a cancer and allows it to grow undetected until it is symptomatic [9, 10] and third, lower levels of BD are associated with a longer preclinical screen-detectable period [11]. Risk prediction algorithms [12, 13] have predominantly used BD as visually assessed by the radiologist according to the Breast Imaging Reporting and Data System (BI-RADS) [14]. Although BI-RADS BD stratifies risk [15], substantial inter-observer variability has generated interest in adopting automated methods [16]. Automated quantitative BD [17–21] would enable more consistent density assessment, and hence, potentially risk assessment for use in breast cancer screening protocols. In addition, automated methods may provide the opportunity to disentangle the most predictive components of BD related to breast cancer risk in a way that visual assessment cannot. For example, quantitative methods can assess whether the absolute or relative amount of BD on mammography (or a combination) represent the key elements that confer breast cancer risk. These algorithms may also be able to separate the risk of breast cancer from the risk of masking, an important distinction when considering the utility of more frequent mammography screening versus the addition of supplemental screening modalities like MRI or ultrasound [22]. Furthermore, few studies have investigated the ability of quantitative BD analysis to predict the risk of interval [23] or advanced cancers [19].

To fill this gap in the literature, we compared women diagnosed with cancer (interval, node-positive, and screen-detected) to disease-free women with respect to BD. We measured BD using automated BD assessments and radiologists’ quantitative visual BD assessments to compare the predictive ability of each BD assessment method. We hypothesised that quantitative BD can predict interval cancers and node-positive screen-detected cancers in order to serve as an imaging biomarker with the potential to personalise breast cancer screening.

## Methods

Ethical approval for the establishment and use of the OPTIMAM image database [24] was obtained from the NHS National Research Ethics Service.

### Study population

In the National Health Breast Screening Programme (NHSBSP), women aged 50–70 years are invited for screening every three years, with an age extension being piloted in a randomised controlled trial of women 47–73 years conducted from 2009 to 2022 [25]. We specifically selected women aged 47–73 who underwent mammographic screening between May 2011 and March 2016 at the Jarvis Breast Screening Centre (Guildford, Surrey, UK) and otherwise met the inclusion criteria for the study. The NHSBSP in general, and the Jarvis Breast Screening Centre specifically, started to convert to digital mammography in 2011. We conducted a retrospective case–control study using mammographic screening images and associated pathological data that were collected as part of the research image database called the OPTIMAM Image Database [24]. Because adherence in a screening program is never exactly within the prescribed round length, due to patient or programme factors (e.g. delayed invitations) we allowed interval cancers to include those found between screening, regardless of timing. This definition means interval cancers are equivalent to post-screening symptomatic cancers in our analysis.

The images were acquired on five Hologic Selenia systems, two Hologic Selenia Dimensions systems (Hologic Inc., Bedford, USA), one GE Senographe Essential system (GE Healthcare Inc., Chicago, USA) and one Sectra MDM-L30 (Phillips Healthcare, Cambridge, Massachusetts, USA). All the digital mammograms in the study were de-identified. Both unprocessed and processed images were collected, when available. To be included in the study, women needed at least one negative digital mammogram prior to the screening mammogram that detected their cancer or the diagnostic mammogram that diagnosed their interval cancer. For the screen-detected cancers, the prior mammogram was used in the study in order to provide an assessment, whether by the radiologist or quantitative imaging, that was ‘blind’ to the cancer. Selection of controls for each case followed a prescribed protocol. Cancer free controls were selected based on the same equipment and ‘date of acquisition’ as the cases. For screen-detected cases, ‘date of acquisition’ was the date of screening examination at which time the cancer is detected. For interval cancers, there were no screening images for detection of cancer (by definition), so ‘date of acquisition’ was date of prior screening images for that individual. From the group of controls meeting these requirements for each case (machine and ‘date of acquisition’), the closest available age was selected. This resulted in 99.4% of cases and controls being within 4 years of age. Because of the limited normal cases in the OPTIMAM database at the time of case/control selection, a one-to-one match protocol was not possible for all. In total, 542 cases had matched controls and 57 cases did not. Thus 63 unmatched controls were included. Matching on other characteristics (e.g. ethnicity or BMI) was not possible because such variables were not available. All the controls were followed up and remained cancer free for at least 3 years. Pathological data were collected from England’s National Breast Screening System.

We required adequate statistical power for comparison of controls with two specific subgroups of cases: interval cancers and node-positive cancers. For both these case groups, we posited that ~20% of controls and 30% of cases would be in the highest density category. Estimating that the total number of controls would be at least double the number of cases in either of these subgroups, 291 cases would give 90% power and 216 cases would give 80% power. We, therefore, aimed to have at least 216 cases in each subgroup. Anticipating that, for some cases and controls, the unprocessed mammograms might not be available, we obtained 599 cases in total, comprising 302 screen-detected cancers and 297 interval cancers. We sought to enrich the dataset for node-positive cases, so all available node-positive cases (*n* = 239) were selected, and node-negative cases (*n* = 360) were selected randomly to complete the case set.

### Breast density assessment

Automated BD software (Volpara Health Technologies Ltd: Version 1.5.1, New Zealand) was used to calculate fibroglandular volume (FGV) in cm^3^, volumetric breast density (VBD) in percent and 5th Edition Volpara Density Grade (DG) from the unprocessed images on the exam level. Volpara is a FDA-approved fully automated software to estimate volumetric breast density [26], based on a detailed relative physics model whereby a region of the breast which is entirely fatty tissue is identified and used as a reference to then calculate the thickness of fibroglandular tissue at each pixel of the image [27]. A model of the breast under compression and the breast thickness (from the DICOM header) are used to convert these fibroglandular tissue thicknesses to volumes, which are then summed across the breast. to provide the FGV and VBD per image. For each screening exam (i.e. a typical four-view exam comprises of the left and right, cranio-caudal (CC) and mediolateral oblique (MLO) views), Volpara software aggregates the image-level metrics to output study-level results per exam. For each breast side, FGV and VBD are averaged across the two views (i.e. CC and MLO), to provide per-breast results for the left and right breasts separately. The study-level FGV and VBD were calculated as the mean of the two per-breast results.

In addition, VBDmax is calculated as the denser VBD of the left or right breasts. Volpara software uses preset cut-off points of VBDmax (to mimic BI-RADS 5th Edition) and reports a study-level 5th Edition Volpara Density Grade (DG), where DG a: 0 ≤ VBD < 3.5%, DG b: 3.5% ≤ VBD < 7.5%, DG c: 7.5 ≤ VBD < 15.5%, DG d: VBD ≥ 15.5%). Typically, the Volpara Density Grades are denoted as VDG a/b/c/d. However, to avoid confusion between acronyms that designate ‘V’ as ‘volume’ or ‘volumetric’ the acronym DG is used throughout this paper, rather than VDG. Volpara software has been validated [26] and used extensively [28] by other groups.

A radiologist (ESB), blinded to case–control status, was shown the images using MedXViewer [29] and assessed BD on a visual analogue scale (VAS) from 0 to 100 for each exam following guidance in prior literature [28].

### Statistical analysis

We took the continuous variables (FGV, VBD and VAS) and determined categorical quartiles using thresholds determined by the distribution for all cases and controls combined (excluding those missing raw images). DG is a categorical variable, already divided by the Volpara software into categories with pre-determined thresholds. We then estimated how these four categorical measures of BD (FGV-quartile, VBD-quartile, VAS-quartile and DG) and how three continuous BD measures (FGV, VBD and VAS) discriminated between cases and controls. We estimated the effects of these BD variables on risk of cancer overall and on the risk of particular subsets of cancers (node-positive, node-negative, interval, and screen-detected) using logistic regression, adjusting for age. For each subgroup of cases, we used all controls as the comparator group.

In addition, we carried out receiver operating characteristic (ROC) analysis, by estimating and comparing areas under the ROC curve (AUCs). We used the De Long et al. [30] method to compare AUCs between BD measures. We compared AUCs between different cases subgroups using permutation tests [31]. Finally, we also provide a, perhaps, more clinically relevant, measure of discrimination showing the numbers in the lowest risk 25% (1st quartile) and the highest risk 25% (4th quartile) of each ‘type’ of cancer.

## Results

### Data description

Our study included 1204 subjects (599 cancers, 605 controls) in women aged 47–73 years old. Dates of mammograms included in this study ranged from 2010 to 2015 (Table 1). Of note, the mammograms included our study for the screen-detected cancers was the prior mammogram. Thus, for the women (defined by the inclusion criteria) who underwent screening between 2011 and 2016 and had a screen-detected cancer, as mentioned in the ‘Methods’ section, the prior mammogram was therefore dated earlier than the inclusion criteria range. For the 302 screen-detected cancers, the time between prior screening exam and diagnosis, as defined by first positive biopsy, was an average 1067 days (range: 454–1196). For the 297 interval cancers, the time between screening and diagnosis was an average of 656 days (range: 26–1991). As expected, a higher proportion of screen-detected than interval cancers were in situ, and a higher proportion of interval cancers were node-positive. The 599 cancers in our study included 524 invasive and 75 cases of ductal carcinoma in situ (DCIS)—for more detail, see Supplementary Information, Supplementary Tables 1 and 2.

**Table 1:** Description of the study population and cancer cases.

		Control		Screen-detected		Interval	
		#	(%)	#	(%)	#	(%)
Mammograms		*N* = 605		*N* = 302		*N* = 297	
Age							
	47–49	37	6.1	14	4.6	22	7.4
	50–54	122	20.2	63	20.9	68	22.9
	55–59	126	20.8	62	20.5	62	20.9
	60–64	154	25.5	79	26.2	63	21.2
	65–69	138	22.8	76	25.2	64	21.5
	70–73	28	4.6	8	2.6	18	6.1
Date of ‘prior’ mammogram							
	2010	27	4.5	12	4	17	5.7
	2011	84	13.9	46	15.2	41	13.8
	2012	292	48.3	204	67.5	81	27.3
	2013	138	22.8	38	12.6	101	34
	2014	51	8.4	2	0.7	45	15.2
	2015	13	2.1	0	0	12	4
Machine							
	Hologic	593	98.0	296	98.0	288	97.0
	GE	9	1.5	6	2.0	9	3.0
	Sectra	3	0.5	0	0	0	0
Invasive/In situ							
	Invasive			245	81.1	279	93.9
	In situ			57	18.9	18	6.1
Nodal status							
	Positive			116	38.4	123	41.4
	Negative			186	61.6	174	58.6
Number of nodes positive							
	None			186	61.6	174	58.6
	1, 2 or 3			101	33.4	89	30
	4 or more			15	5	34	11.4

### Categorical quantitative BD predicting cancer types

Unprocessed images needed for automated BD measures were available for 429 (72%) cases and 418 (69%) controls. FGV-quartile, VAS-quartile, and DG predicted all cancers versus controls, while VBD-quartile did not (Table 2). The steepest risk gradient for all cancers was associated with FGV with an odds ratio (OR) for the highest quartile compared to the lowest of 3.7 (95% CI 2.5–5.6).

**Table 2:** Association of categorical measures of density with cancer risk (all cancers).

	Controls		All cancers				
	#	%	#	%	OR	95% CI	*p*-value
FGV (cm^3^)^a^							
1st quartile	137	22.6	75	12.5	1		*p* < 0.01
2nd quartile	114	18.8	98	16.4	1.6	(1.1, 2.3)	
3rd quartile	95	15.7	116	19.4	2.3	(1.5, 3.4)	
4th quartile	72	11.9	140	23.4	3.7	(2.5, 5.6)	
Missing	187	30.9	170	28.4			
VBD (%)^b^							
1st quartile	119	19.7	100	16.7	1		*p* = 0.12
2nd quartile	107	17.7	99	16.5	1.1	(0.7, 1.6)	
3rd quartile	101	16.7	111	18.5	1.3	(0.9, 1.9)	
4th quartile	91	15.0	119	19.9	1.6	(1.0, 2.3)	
Missing	187	30.9	170	28.4			
VAS (%)^c^							
1st quartile	174	28.8	143	23.9	1		
2nd quartile	157	26	137	22.9	1.1	(0.8, 1.5)	*p* = 0.04
3rd quartile	132	21.8	165	27.5	1.5	(1.1, 2.1)	
4th quartile	142	23.5	154	25.7	1.3	(0.9, 1.8)	
DG							
1	27	4.5	14	2.3	1		*p* = 0.04
2	206	34.0	193	32.2	1.7	(0.9, 3.5)	
3	135	22.3	151	25.2	2.1	(1.0, 4.2)	
4	50	8.3	71	11.9	2.6	(1.3, 5.7)	
Missing	187	30.9	170	28.4			

Quartile cut-points.

^a^FGV: 11.70, 37.95, 51.30, 73.35, 306.50.

^b^VBD: 2.4, 4.8, 6.9, 10.9, 30.0.

^c^VAS: 1.9, 29.0, 47.0, 64.0, 96.1.

**Fig. 1:**
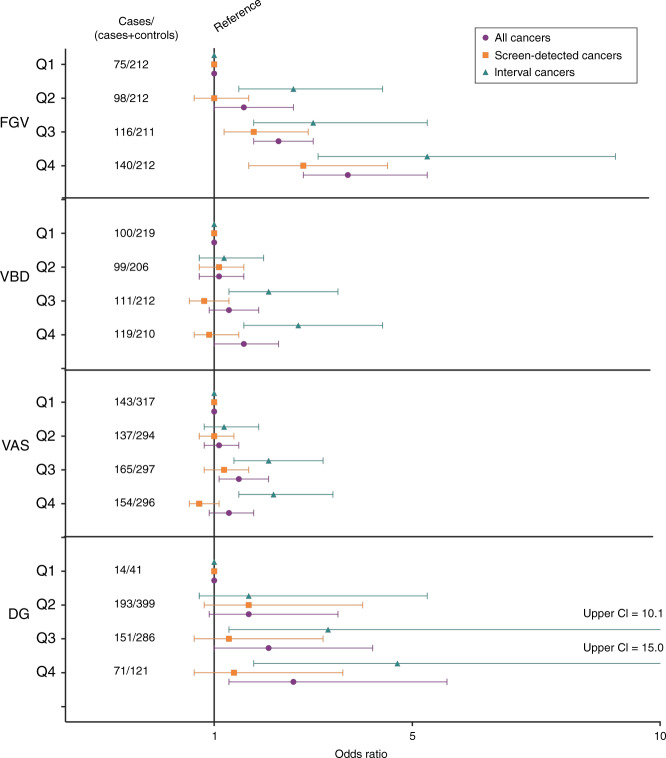
Visual depiction of BD quartile ability to discriminate all, screen-detected, and interval cancers. Associations between categorical mammographic measures of breast density and breast cancer risk are described by odds ratios for all cancers, screen-detected and interval cancers as compared to controls.

VAS-quartile was not associated with node-positive cancers. In contrast, all categorical automated BD predicted interval cancers and ‘node-positive or interval’ cancers (henceforth referred to as ‘combined’ cancers) with statistical significance (Table 3). FGV-quartile, VBD-quartile, and DG statistically significantly predicted node-positive cancers. FGV-quartile demonstrated the steepest risk gradient for interval (OR 5.3, CI 3.1, 9.1, *p* < 0.01), node-positive (OR 4.7, CI 2.5, 9.0, *p* < 0.01) and combined cancers (OR 4.7, CI 2.9, 7.8, *p* < 0.01). All automated BD measures more consistently predicted interval compared to screen-detected cancers (Fig. 1)—for more detail, see Supplementary Information, Supplementary Table 3.

**Table 3:** Association of categorical measures of density with risk of interval, node-positive, and combined (interval or node-positive).

	Controls		Interval cancers					Node-positive cancers					Combined				
	#	%	#	%	OR	95% CI	*p*-value	#	%	OR	95% CI	*p*-value	#	%	OR	95% CI	*p*-value
FGV (cm^3^)																	
1st quartile	137	22.6	26	8.8	1		*p* < 0.01	17	7.1	1		*p* < 0.01	34	8.2	1		*p* < 0.01
2nd quartile	114	18.8	56	18.9	2.6	(1.5, 4.4)		34	14.2	2.4	(1.3, 4.6)		66	16.0	2.3	(1.4, 3.8)	
3rd quartile	95	15.7	55	18.5	3	(1.8, 5.3)		31	13.0	2.6	(1.4, 5.1)		63	15.3	2.7	(1.6, 4.4)	
4th quartile	72	11.9	72	24.2	5.3	(3.1, 9.1)		42	17.6	4.7	(2.5, 9.0)		84	20.3	4.7	(2.9, 7.8)	
Missing^a^	187	30.9	88	29.6				115	48.1				166	40.2			
VBD (%)																	
1st quartile	119	19.7	35	11.8	1		*p* < 0.01	24	10.0	1		*p* = 0.02	46	11.1	1		*p* < 0.01
2nd quartile	107	17.7	37	12.5	1.2	(0.7, 2.0)		27	11.3	1.2	(0.7, 2.3)		47	11.4	1.1	(0.7, 1.8)	
3rd quartile	101	16.7	65	21.9	2.1	(1.3, 3.5)		29	12.1	1.4	(0.8, 2.6)		73	17.7	1.8	(1.1, 2.9)	
4th quartile	91	15.0	72	24.2	2.7	(1.6, 4.4)		44	18.4	2.3	(1.3, 4.2)		81	19.6	2.3	(1.4, 3.6)	
Missing^a^	187	30.9	88	29.6				115	48.1				166	40.2			
VAS (%)																	
1st quartile	174	28.8	53	17.8	1		*p* < 0.01	55	23	1		*p* = 0.14	86	20.8	1		*p* < 0.01
2nd quartile	157	25.9	59	19.9	1.2	(0.8, 1.9)		55	23	1.1	(0.7, 1.7)		89	21.6	1.1	(0.8, 1.6)	
3rd quartile	132	21.8	85	28.6	2.1	(1.4, 3.2)		63	26.4	1.5	(1.0, 2.3)		116	28.1	1.8	(1.2, 2.5)	
4th quartile	142	23.5	100	33.7	2.2	(1.5, 3.4)		66	27.6	1.5	(1.0, 2.3)		122	29.5	1.7	(1.2, 2.4)	
DG																	
1	27	4.5	5	1.7	1		*p* < 0.01	4	1.7	1		*p* = 0.01	6	1.5	1		*p* < 0.01
2	206	34.0	70	23.6	1.7	(0.7, 5.3)		48	20.1	1.5	(0.6, 5.4)		91	22.0	1.9	(0.8, 5.2)	
3	135	22.3	88	29.6	3.3	(1.3, 10.1)		44	18.4	2.1	(0.8, 7.5)		99	24.0	3.1	(1.3, 8.6)	
4	50	8.3	46	15.5	4.7	(1.8, 15.0)		28	11.7	3.6	(1.2, 13.1)		51	12.3	4.4	(1.7, 12.6)	
Missing^a^	187	30.9	88	29.6				115	48.1				166	40.2			

^a^Missing applies to all density measures except VAS.

### Continuous BD measures predicting cancer types

For continuous BD measures (FGV, VBD and VAS), the differences in means between cases and controls were statistically significant for all, interval, node-positive, and combined cancers (Table 4). The difference in means for FGV between cases and controls was statistically significant for screen-detected cancers. The difference in means for FGV and the difference in means for VAS between cases and controls were statistically significant for node-negative cancers.

**Table 4:** Associations of all cancers, screen-detected, interval, node-negative, node-positive and combined (node-positive or interval) cancers with continuous breast density measures.

	Mean	Mean	Difference	CI	*p*-value	AUC	95% CI^a^	*p*-value^b^
	Controls	All cancers						*p* < 0.01
FGV (cm^3^)	53.7	66.3	12.6	(8.1, 17.1)	*p* < 0.01	0.63	(0.59, 0.67)	
VBD (%)	8.2	9.2	1.0	(0.3, 1.7)	*p* < 0.01	0.56	(0.51, 0.60)	
VAS (%)	44.4	48.2	3.8	(1.3, 6.4)	*p* < 0.01	0.55	(0.51, 0.59)	
	Controls	Screen-detected						*p* < 0.01
FGV (cm^3^)	53.7	64.4	10.8	(5.0, 16.5)	*p* < 0.01	0.61	(0.56, 0.66)	
VBD (%)	8.2	8.1	−0.1	(−0.9, 0.8)	*p* = 0.87	0.51	(0.46, 0.56)	
VAS (%)	44.4	44.1	−0.3	(−3.3, 2.7)	*p* = 0.84	0.50	(0.46, 0.55)	
	Controls	Interval cancers						*p* = 0.06
FGV (cm^3^)	53.7	68.2	14.5	(8.9, 20.1)	*p* < 0.01	0.65	(0.60, 0.70)	
VBD (%)	8.2	10.3	2.1	(1.2, 3.0)	*p* < 0.01	0.63	(0.58, 0.68)	
VAS (%)	44.4	52.4	8.1	(5.0, 11.1)	*p* < 0.01	0.60	(0.56, 0.65)	
	Controls	Node-negative cancers						*p* < 0.01
FGV (cm^3^)	53.7	64.1	10.4	(5.8, 15.0)	*p* < 0.01	0.62	(0.58, 0.67)	
VBD (%)	8.2	8.8	0.6	(−0.2, 1.4)	*p* = 0.13	0.54	(0.49, 0.59)	
VAS (%)	44.4	47.5	3.1	(0.2, 6.0)	*p* = 0.04	0.54	(0.50, 0.58)	
	Controls	Node-positive cancers						*p* < 0.01
FGV (cm^3^)	53.7	71.7	18.0	(9.5, 26.4)	*p* < 0.01	0.65	(0.59, 0.71)	
VBD (%)	8.2	10.1	1.9	(0.7, 3.1)	*p* < 0.01	0.60	(0.54, 0.66)	
VAS (%)	44.4	49.3	4.9	(1.6, 8.2)	*p* < 0.01	0.56	(0.51, 0.61)	
	Controls	Combined						*p* < 0.05
FGV (cm^3^)	53.7	69.2	15.5	(9.8, 21.2)	*p* < 0.01	0.65	(0.60, 0.69)	
VBD (%)	8.2	10.0	1.8	(0.9, 2.7)	*p* < 0.01	0.61	(0.56, 0.66)	
VAS (%)	44.4	50.5	6.2	(3.4, 9.0)	*p* < 0.01	0.58	(0.54, 0.62)	

^a^95% Confidence intervals that do not include 0.50 demonstrate a statistically significantly better discriminatory ability compared to chance.

^b^This *p*-value reflects whether there is a statistically significant difference between the AUCs of the continuous quantitative BD measurements.

**Fig. 2:**
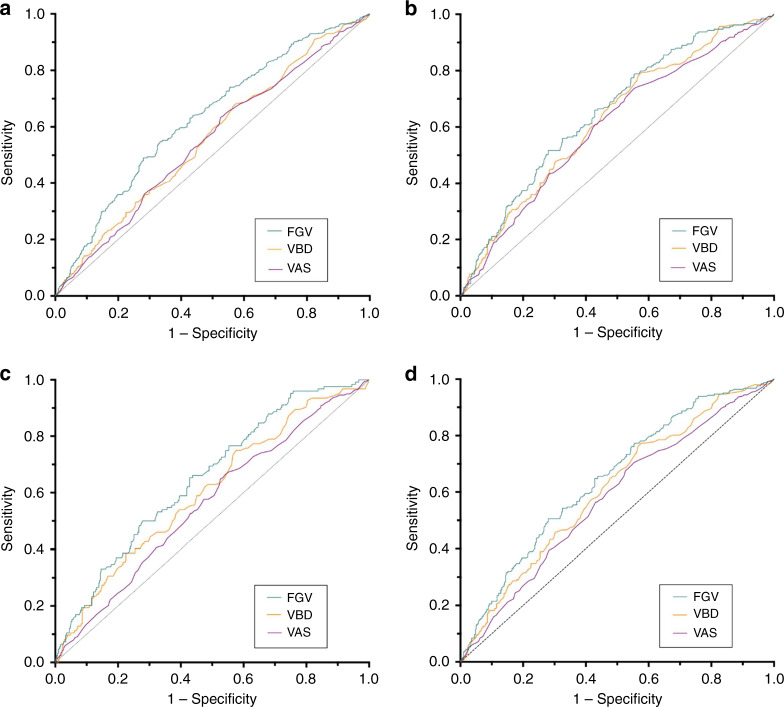
Ability Continuous BD measures to discriminate cancer types. Receiver operating characteristic (ROC) curves for continuous mammographic measures of breast density to discriminate **a** all cancers, **b** interval, **c** node-positive, and **d** combined cancers (interval or node-positive) from controls.

AUC analysis (Fig. 2) demonstrates that FGV reached the highest discriminative ability with an AUC of 0.65 for three subsets of cancers: interval cancers (95% CI 0.60, 0.70), node-positive cancers (95% CI 0.59, 0.71), and combined cancers (95% CI 0.60, 0.69). FGV, VBD and VAS were each able to discriminate all, interval, node-positive, and combined cancers from controls, as demonstrated by AUC 95% confidence intervals not including 0.50 (Table 4). VBD and VAS were not able to discriminate screen-detected cancers from controls.

There were clear differences between the AUCs of the three BD measures for all (*p* < 0.01), node-positive (*p* < 0.01) and combined (*p* = 0.02) cancers, but only a moderate difference between BD measures for interval cancers (*p* = 0.06).

To provide a metric that may be more clinically relevant than AUC, we determined the numbers of each ‘type’ of cancer by risk quartile: the lowest risk 25% (1st quartile) and the highest risk 25% (4th quartile). Results showing the highest risk 25% (4th quartile) for all subcategories of cancers including screen-detected, interval, node-positive, and node-negative demonstrate that FGV captures at least as high a percentage of these cancers as VBD and VAS (Table 5) emulating an exemplar scenario of women who may be candidates for additional screening if the threshold was set below the 4th quartile. FGV categorises at least 10% more screen-detected and node-negative cancers in the highest risk category (4th quartile) as compared VBD and VAS.

**Table 5:** The numbers and percentage of each type of cancer by quantitative density risk quartile.

Density measure	Category	Controls *N* (%)	Screen-detected cancers *N* (%)	Interval cancers *N* (%)	Node + Cancers(%)	Node − Cancers(%)
FGV	1st quartile	137 (32.8)	49 (22.3)	26 (12.4)	17 (13.7)	58 (19)
	2nd & 3rd quartile	209 (50)	103 (46.8)	111 (53.1)	65 (52.4)	149 (48.9)
	**4th quartile**	**72 (17.2)**	**68 (30.9)**	**72 (34.4)**	**42 (33.9)**	**98 (32.1)**
VBD	1st quartile	119 (28.5)	65 (29.5)	35 (16.7)	24 (19.4)	76 (24.9)
	2nd & 3rd quartile	208 (49.8)	108 (49.1)	102 (48.8)	56 (45.2)	154 (50.5)
	**4thquartile**	**91 (21.8)**	**47 (21.4)**	**72 (34.4)**	**44 (35.5)**	**75 (24.6)**
VAS	1st quartile	174 (28.8)	90 (29.8)	53 (17.8)	55 (23)	88 (24.4)
	2nd & 3rd quartile	289 (47.8)	158 (52.3)	144 (48.5)	118 (49.4)	184 (51.1)
	**4thquartile**	**142 (23.5)**	**54 (17.9)**	**100 (33.7)**	**66 (27.6)**	**88 (24.4)**

The highest risk women, 4th quartile is bolded.

FGV, VBD and VAS were all significantly more discriminative of interval cancers than of screen-detected cancers (*p* = 0.04, *p* < 0.01 and *p* < 0.01 respectively). Only VBD was significantly more predictive of node-positive than of node-negative cancers (*p* < 0.01), although all three measures had greater AUCs for node-positive than node-negative cancers. All three automated BD methods showed higher AUCs for (i) interval compared to screen-detected; (ii) combined compared to node-negative; and (iii) combined compared to screen-detected cancers. The AUC for interval cancers was significantly greater than the AUC for screen-detected cancers for VBD (*p* < 0.01) and VAS (*p* < 0.01), and suggestively so for FGV (*p* = 0.07).

## Discussion

FGV significantly discriminated all, interval, screen-detected, node-positive and node-negative cancers compared to controls. VBD, VAS and DG discriminated interval or node-positive cancers but did not consistently discriminate screen-detected or node-negative cancers. The relative discriminative ability of FGV, overall and for each/individual cancer subtypes/groups was either equivalent to or, in most cases, greater than that of VAS or VBD, whether using logistic regression (captured by the steepness of the odds ratio gradient), ROC analysis (captured by AUC), or number of cancers included in the highest risk category (4th quartile). Of note, for VBD and VAS, interval cancer prediction was significantly greater (by AUC) than screen-detected cancer prediction while FGV only showed a statistical trend. This phenomenon underscores the differential ability of FGV to discriminate screen-detected cancers, knowing that FGV has generally higher AUCs for virtually all comparisons (Table 4).

If quantitative breast density is to be successfully used for stratified screening protocols to decrease interval and advanced breast cancers, prediction of both the risk of breast cancer and the risk of masking by mammographic breast density will be important. It stands to reason that screen-detected cancers are less affected by masking because they were detected on mammography and, thus, not sufficiently obscured by dense fibroglandular tissue to preclude detection. On the other hand, interval cancers are likely to be more affected by masking because they were not detected by mammography. However, this relationship between interval cancers and masking is far from perfect because interval cancers may also be related to rapid growth between screening examinations or to an interpretation error. Therefore, screen-detected cancers may map more strongly to breast cancer risk as compared to masking. Correspondingly, interval cancers may map more strongly to masking but also involve a component of breast cancer risk. In our study, because VBD and VAS only discriminate interval or node-positive cancers from controls, these algorithms may correlate more strongly with masking. On the other hand, FGV, which additionally discriminates screen-detected cancers from controls may have an added correlation to breast cancer risk. Perhaps FGV maps to both breast cancer and masking risk by measuring absolute BD volume as compared to VBD and VAS, which measure percent BD. There is a precedent for stronger prediction of breast cancer risk generally from absolute rather than percentage density measures [17, 32]. Results, however, are by no means uniform [28]. There is a need for methodological development to disentangle how absolute versus percent fibroglandular volume map to breast cancer risk and masking.

Our results are comparable to results of the single study that analysed interval cancers in a screening programme with a long screening interval (3 years) and tested several quantitative BD techniques [19]. Wanders et al. found that absolute volume of breast density (FGV) predicted screen-detected cancers whereas percent density by volume (VBD) did not [19]. In this study as with the present work, both FGV and VBD predicted interval cancers. Unlike our study, a study by Kerlikowske and colleagues that evaluated the ability of percent density by volume (VBD) to predict screen-detected cancer in a population with a shorter screening interval (1–2 years) showed predictive ability in both screen-detected and interval cancers, with interval cancer prediction being statistically significantly superior [15]. The differences in these results may be attributable to differences in cancers included in screen versus interval groups when the screening interval is shorter, as is the case in the U.S., with a larger fraction of more aggressive cancers included in the interval group. Women in the US may more frequently be offered supplemental screening, influencing patterns of early detection, or be different in terms of breast cancer risk. For example, in the U.S. study, a high proportion (just under 20%) of controls had a history of breast biopsy [15]. This phenomenon may also relate to the fact that our cases and controls were selected to have the same age distribution, which would remove any density differences between intervals and cancer free controls which were due to confounding with age. Astley et al. [28] found VAS more predictive than the automated measures such as FGV and VBD, but these investigators evaluated images acquired on GE equipment, whereas most images used in our study were acquired on Hologic equipment. The differences in the processed images between these two types of equipment may have affected readers’ VAS estimation of BD. The literature shows that image processing significantly affects cancer detection [33], but more work is needed to confirm if image processing also influences visual BD estimation. Overall, the inability to obtain separate estimates for effects on ‘pure’ masking risk and ‘pure’ breast cancer risk may account for some of the variation in findings between studies.

Whatever the mechanism for measuring BD, women with high levels of BD have an increased risk of interval or node-positive cancers, motivating the need to augment the screening regimen. Women at high breast cancer risk but not at high masking risk, may benefit from increased mammography screening frequency. Women at high masking risk only or high cancer and masking risk, may be better served by screening with modalities supplementary to mammography, like MRI or ultrasound. In fact, there is interest in determining and targeting these different opportunities for improved screening outcomes (masking versus breast cancer risk) and modelling these strategies [34]. In our study, we find that FGV discriminates all categories of cancer more strongly than other density measures perhaps capturing masking and risk more fully than VBD, VAS and DG, which only discriminate interval and node-positive cancers from controls.

The strengths of our study include our assessment of the discriminative ability of several measures of BD and risk of breast cancer. We also provide an important analysis of volumetric BD related to interval cancer risk [15, 19, 35, 36] and the first related to node-positive cancers. Our cases and controls were selected to have as similar an age distribution as possible, which would remove any density differences between intervals and cancer free controls which were due to confounding by age. However, because density as a risk factor is conditional on age (hence our design and analysis) comparing risks for two women of different ages based on density is not possible based on our work. To fully utilise the risk dimension of density in a screening program, further investigation will be required. For example, a large series of unselected mammograms could be used to construct age-specific reference ranges for density, which would then be a foundation to further refine screening practice.

We did not collect detailed information in relation to a number of covariates (demographic, hormonal, reproductive, lifestyle and family history). We also did not have BMI, which is known to improve discriminatory capacity of quantitative BD measurements [37]. As expected, the time between the analysed mammograms (the most recent normal) for the screen-detected cases was longer than the time between the analysed mammogram and the interval cancers; an unavoidable difference based on the realities of a population-based breast cancer screening programme. This difference raises the question whether adjustment for this difference; i.e. adjustment beyond age may be necessary. We have carried out several major re-analyses incorporating adjustment for time since prior mammogram for those in whom individual matching was possible, revealing no substantive changes to our results or conclusions. Finally, some cases and controls did not have unprocessed images, and thus the quantitative BD measures were not calculated in these patients. However, our a *priori* power calculation anticipated these missing images, which therefore should not have influenced our results or conclusions.

We find that FGV has the potential to predict the important components of risk that may provide the foundation for stratified screening: risk of cancer, risk of aggressive cancer, and risk of masking effects. While any quantitative BD measure will undoubtedly be one variable among many predictive variables that will contribute to decisions about breast cancer screening, we believe that our analysis adds to the literature that will inform a more comprehensive model to be tested in the future. Our findings suggest that FGV may be a comparatively better imaging biomarker suited to provide guidance for more intensive stratified screening for mammography, such as a shortened screening interval. VBD, VAS and DG, by predominantly predicting interval cancers and node-positive cancers may selectively correlate with masking risk and be more suited to directing women to supplemental screening modalities other than mammography.

## Supplementary information

Supplementrary Information

## Data Availability

Mammographic screening images and associated pathological data that were collected as part of the research image database called the OPTIMAM Mammography Image Database cited in the text of the manuscript methods section. The OPTIMAM Mammography Image Database, funded by Cancer Research UK, used in the current study are available and can be found here https://medphys.royalsurrey.nhs.uk/omidb/.
