# Supplementary material for: Quantitative breast density analysis to predict interval and node-positive cancers in pursuit of improved screening protocols: a case–control study
Source: Br J Cancer. 2021 Jun 24;125(6):884–92. doi: 10.1038/s41416-021-01466-y (PMC8438060; doi:10.1038/s41416-021-01466-y)
Supplement: Supplementary file 1 — Supplementrary Information [file 41416_2021_1466_MOESM1_ESM.docx]

**SUPPLEMENTARY INFORMATION**

***Further Detail on Study Subject Demographics***

For invasive cancers, a greater proportion of interval cancers were grade 3 compared with screen-detected (Supplementary Information Table 1).

**Supplementary Table 1:** Description of Invasive cancer cases

|  |  | **Screen-detected** | | **Interval** | |
| --- | --- | --- | --- | --- | --- |
|  |  | **#** | **(%)** | **#** | **(%)** |
| **Mammograms** | | N=245 |  | N=279 |  |
| **Invasive Grade** | |  |  |  |  |
|  | **G1** | 45 | 18.4 | 26 | 9.3 |
|  | **G2** | 150 | 61.2 | 140 | 50.2 |
|  | **G3** | 47 | 19.2 | 93 | 33.3 |
|  | **Missing** | 3 | 1.2 | 20 | 7.2 |
| **Cancer type** | |  |  |  |  |
|  | **ductal** | 196 | 80 | 221 | 79.2 |
|  | **lobular** | 30 | 12.2 | 37 | 13.3 |
|  | **mixed** | 10 | 4.1 | 6 | 2.2 |
|  | **special** | 9 | 3.7 | 15 | 5.4 |

DCIS cases, whether diagnosed as screen-detected or interval cancers, did not differ significantly by grade, however only a small numbers of DCIS cases were diagnosed as interval cancers (Supplementary Information Table 2).

**Supplementary Table 2:** Description of DCIS cases

|  |  | **Screen-detected** | | **Interval** | |
| --- | --- | --- | --- | --- | --- |
|  |  | **#** | **(%)** | **#** | **(%)** |
| **Mammograms** | | N=57 |  | N=18 |  |
| **In situ Grade** | |  |  |  |  |
|  | **High** | 31 | 54.4 | 11 | 61.1 |
|  | **Intermediate** | 21 | 36.8 | 5 | 27.8 |
|  | **Low** | 3 | 5.3 | 1 | 5.6 |
|  | **Missing** | 2 | 3.5 | 1 | 5.6 |

***Further Detail on Screen Detected and Node-negative Cases***

FGV-quartile predicted screen-detected (p<0.01) and node-negative (p<0.01) cancers while VBD-quartile, VAS-quartile and DG did not (Supplementary Information Table 3).

**Supplementary Table 3:** Effect of categorical measures of density on risk of screen-detected and node-negative cancer

|  | **Controls** | | **Screen-detected cancers** | | | | | **Node-negative cancers** | | | | |
| --- | --- | --- | --- | --- | --- | --- | --- | --- | --- | --- | --- | --- |
|  | # | % | # | % | OR | 95% CI | p-value | # | % | OR | 95% CI | p-value |
| **FGV (cm^3^)** |  |  |  |  |  |  |  |  |  |  |  |  |
| 1st quartile | 137 | 22.6 | 49 | 16.2 | 1 |  | p<0.01 | 58 | 16.1 | 1 |  | p<0.01 |
| 2nd quartile | 114 | 18.8 | 42 | 13.9 | 1 | (0.6, 1.7) |  | 64 | 17.8 | 1.3 | (0.9, 2.1) |  |
| 3rd quartile | 95 | 15.7 | 61 | 20.2 | 1.8 | (1.2, 2.9) |  | 85 | 23.6 | 2.2 | (1.4, 3.3) |  |
| 4th quartile | 72 | 11.9 | 68 | 22.5 | 2.8 | (1.7, 4.5) |  | 98 | 27.2 | 3.4 | (2.2, 5.3) |  |
| Missing | 187 | 30.9 | 82 | 27.2 |  |  |  | 55 | 15.3 |  |  |  |
| **VBD (%)** |  |  |  |  |  |  |  |  |  |  |  |  |
| 1st quartile | 118 | 19.5 | 65 | 21.5 | 1 |  | p=0.77 | 76 | 21.1 | 1 |  | p=0.55 |
| 2nd quartile | 107 | 17.7 | 62 | 20.5 | 1.1 | (0.7, 1.6) |  | 72 | 20.0 | 1 | (0.7, 1.6) |  |
| 3rd quartile | 101 | 16.7 | 46 | 15.2 | 0.8 | (0.5, 1.3) |  | 82 | 22.8 | 1.2 | (0.8, 1.9) |  |
| 4th quartile | 91 | 15.0 | 47 | 15.6 | 0.9 | (0.6, 1.5) |  | 75 | 20.8 | 1.3 | (0.8, 2.0) |  |
| Missing | 188 | 31.1 | 82 | 27.2 |  |  |  | 55 | 15.3 |  |  |  |
| **VAS (%)** |  |  |  |  |  |  |  |  |  |  |  |  |
| 1st quartile | 174 | 28.8 | 90 | 29.8 | 1 |  | p=0.18 | 88 | 24.4 | 1 |  | p=0.09 |
| 2nd quartile | 157 | 26 | 78 | 25.8 | 1 | (0.7, 1.4) |  | 82 | 22.8 | 1 | (0.7, 1.5) |  |
| 3rd quartile | 132 | 21.8 | 80 | 26.5 | 1.2 | (0.8, 1.7) |  | 102 | 28.3 | 1.5 | (1.1, 2.2) |  |
| 4th quartile | 142 | 23.5 | 54 | 17.9 | 0.7 | (0.5, 1.1) |  | 88 | 24.4 | 1.2 | (0.8, 1.8) |  |
| **DG** |  |  |  |  |  |  |  |  |  |  |  |  |
| 1 | 27 | 4.5 | 9 | 3.0 | 1 |  | p=0.37 | 10 | 2.8 | 1 |  | p=0.23 |
| 2 | 206 | 34.0 | 123 | 40.7 | 1.7 | (0.8, 4.0) |  | 145 | 40.3 | 1.8 | (0.9, 4.0) |  |
| 3 | 135 | 22.3 | 63 | 20.9 | 1.3 | (0.6, 3.2) |  | 107 | 29.7 | 2 | (1.0, 4.6) |  |
| 4 | 50 | 8.3 | 25 | 8.3 | 1.4 | (0.6, 3.6) |  | 43 | 11.9 | 2.2 | (1.0, 5.4) |  |
| Missing | 187 | 30.9 | 82 | 27.2 |  |  |  | 55 | 15.3 |  |  |  |
